# Diversity, distribution, and significance of transposable elements in the genome of the only selfing hermaphroditic vertebrate *Kryptolebias marmoratus*

**DOI:** 10.1038/srep40121

**Published:** 2017-01-10

**Authors:** Jae-Sung Rhee, Beom-Soon Choi, Jaebum Kim, Bo-Mi Kim, Young-Mi Lee, Il-Chan Kim, Akira Kanamori, Ik-Young Choi, Manfred Schartl, Jae-Seong Lee

**Affiliations:** 1 Department of Marine Science, College of Natural Sciences, Incheon National University, Incheon 22012, South Korea; 2 Phyzen Genomics Institute, Seoul 08787, South Korea; 3 Department of Stem Cell and Regenerative Biology, College of Animal Bioscience & Technology, Konkuk University, Seoul 05029, South Korea; 4 Department of Biological Science, College of Science, Sungkyunkwan University, Suwon 16419, South Korea; 5 Department of Life Science, College of Natural Sciences, Sangmyung University, Seoul 03016, South Korea; 6 Division of Polar Life Sciences, Korea Polar Research Institute, Incheon 21990, South Korea; 7 Division of Biological Science, Graduate School of Science, Nagoya University, Furocho Chikusa, Nagoya 464-8602, Japan; 8 Department of Agriculture and Life Industry, Kangwon National University, Chuncheon 24341, South Korea; 9 Physiological Chemistry, University of Würzburg, Biozentrum, Am Hubland, and Comprehensive Cancer Center, University Clinic Würzburg, Josef Schneider Straße 6, 98074 Würzburg, Germany, and Institute for Advanced Studies and Department of Biology, Texas A&M University, College Station, Texas 77843, USA

## Abstract

The *Kryptolebias marmoratus* is unique because it is the only self-fertilizing hermaphroditic vertebrate, known to date. It primarily reproduces by internal self-fertilization in a mixed ovary/testis gonad. Here, we report on a high-quality genome assembly for the *K. marmoratus* South Korea (SK) strain highlighting the diversity and distribution of transposable elements (TEs). We find that *K. marmoratus* genome maintains number and composition of TEs. This can be an important genomic attribute promoting genome recombination in this selfing fish, while, in addition to a mixed mating strategy, it may also represent a mechanism contributing to the evolutionary adaptation to ecological pressure of the species. Future work should help clarify this point further once genomic information is gathered for other taxa of the family Rivulidae that do not self-fertilize. We provide a valuable genome resource that highlights the potential impact of TEs on the genome evolution of a fish species with an uncommon life cycle.

Reproduction by selfing is a common phenomenon in plants and many hermaphroditic invertebrates, but it has not been detected in vertebrates^1^ except for the extraordinary mangrove killifishes (*Kryptolebias marmoratus* and closely related forms). This species routinely reproduces by self-fertilization^2^,^3^. Eggs are internally fertilized by sperm that is produced in the testicular part of the bisexual composite gonad^4^. Selfing of the mangrove killifish leads to high degree of inbreeding and genome homogenization^3^. Natural populations of *K. marmoratus* are often inbred to the extent that may be viewed as assemblages of clonal lineages that are genetically variable^5^,^6^,^7^. However, occasional outcrossing with males is possible that may also contribute to the species’ capacity to overcome ecological pressure^7^. Historically, *K. marmoratus* had been considered as a single species with an enormous geographic range (Florida to southeastern Brazil). At a broader phylogeographic scale, *K. marmoratus* was found to be comprised by at least two genetically and geographically distinct lineages^8^. The Panama (PAN-RS) and Dangriga, Belize (DAN) are strains that represent each lineage^8^. Recently, a restriction site-associated DNA (RAD)-seq linkage map was made from a hybrid between these strains (PAN-RS was referred to *K. hermaphroditus* and Dan, *K. marmoratus* therein) utilizing significant genetic differences between them^9^. Since extensive phylogeographic analyses of the *K. marmoratus* ‘species complex’ are being undergone but are still ambiguous to determine consensus on the level of taxonomic recognition, we conservatively refer it as the ‘South Korea (SK) strain’ and do not assign it to any particular species.

Although *K. marmoratus* consists of androdioecious populations, the mixed mating strategy, composed of dominant selfing and occasional outcrossing with gonochoristic males^10^ has puzzled biologists on the adaptive significance of such systems. In *K. marmoratus*, there are two types of males that have been observed. First, primary males have functional testicular but not ovarian tissues. Such males were found to occur, although rarely, in nature, and can be induced, under certain conditions, in the laboratory^11^. Second, males are typically the result of hermaphrodites that transform into secondary males by ovarian atresia^11^. Most of the males in natural populations of this kind have been found to have transformed into an early life stage thereby ovarian tissue is typically absent at later life^5^,^12^,^13^,^14^,^15^. High levels of inbreeding like those observed in *K. marmoratus* are considered as maladaptive for a number of reasons like for example susceptibility to diseases^16^,^17^. Nevertheless, the evolutionary forces that are key for maintaining the predominantly selfing reproductive mode and limiting mixed mating still remain largely unclear.

Mangrove killifish are easily kept and maintained in the laboratory. As a laboratory model, mangrove killifish offers most advantages of fish models [comparable to zebrafish (*Danio rerio*) and medaka (*Oryzias latipes*)] including transparent embryos, breeding in large numbers and the ability to produce embryos from artificial insemination^18^,^19^. This is combined with its unique feature of isogenicity. Together these characters make the mangrove killifish a suitable model organism for environmental toxicology as well as for the understanding of the evolution of phenotypic plasticity^20^. To make full use of an emerging model system and to understand the unique features of the mangrove killifish, including its physiological plasticity and the evolution and effects of selfing reproduction in a vertebrate, the availability of a high-quality reference genome is required. Recently, approximately 900 Mb of the genome sequence, including 27,328 protein-coding genes of the *K. marmoratus* Reckley Hill Lake (RHL), Bahamas strain, was published^21^. Here, we report the genome assembly and annotation of the SK strain of mangrove killifish and its analysis. We compare both genomes and present evidence on the utility of transposable elements (TEs) as a molecular mechanism of the mangrove killifish for evolutionary adaptation mechanism to ecological pressure.

## Results

The haploid genome of the mangrove killifish is encoded on 24 chromosomes^22^. We sequenced the genome of the SK strain of mangrove killifish by employing a whole genome shotgun approach to 118 x read coverage and 2,418 x physical coverage (estimated genome size of 680 Mbs; Suppl. Fig. 1) using the Illumina HiSeq 2000 platform. We prepared five pair-end libraries spanning insert sizes from 200 bp to 20 kb (Suppl. Table 1). A total of 80 Gb of sequence data were generated from paired-end reads. The inbreeding-mediated isogenic genome of *K. marmoratus* facilitated the *de novo* assembly. Genome assembly using ALLPATHS-LG (ver. r42411; Table 1) was performed to produce scaffolds, yielding finally 3,072 scaffolds with N50 length of 2.2 Mb (Table 1). The total length of scaffolds is 680 Mb, which is consistent with *K*-mer prediction. Assuming conserved synteny with the closest phylogenetic relative, the medaka, we employed a ‘reference-assisted’ assembly strategy^23^ that improved the assembly by allowing to build larger scaffolds after correcting for misassemblies (Suppl. Table 2). By comparison, the number of genome assembly statistics became higher compared to that of the RHL strain. The genome assembly of the RHL strain of mangrove killifish resulted in 7,929 scaffolds ( > 10 kb) with N50 length of 112 kb^21^.

Quality of the assembly was assessed by a core eukaryotic gene mapping method (Suppl. Table 3). Also, the intactness of large-scale gene clusters such as *Titin A/B*, major histocompatibility complex (*MHC*) class I, and homeobox (*Hox*) gene family clusters strongly confirmed that the genome assembly of *K. marmoratus* is of high quality (Suppl. Figs 2, 3 and 4). Gene level synteny comparison with other teleost genomes (e.g. medaka, stickleback, and zebrafish) also showed highly conserved gene content in mangrove killifish scaffolds subject to phylogenetic distance discrepancies, as for example the mangrove killifish is evolutionary more closely related to medaka than to zebrafish thereby the fraction of scaffolds with breakpoints is expected to increase for zebrafish (Suppl. Fig. 5). The GC content was 38% based on 500 bp non-overlapping sliding window along the genome assemblies (Suppl. Fig. 6). The ratio is comparable to the GC content of the RHL strain genome (39%)^21^.

**Figure 1:**
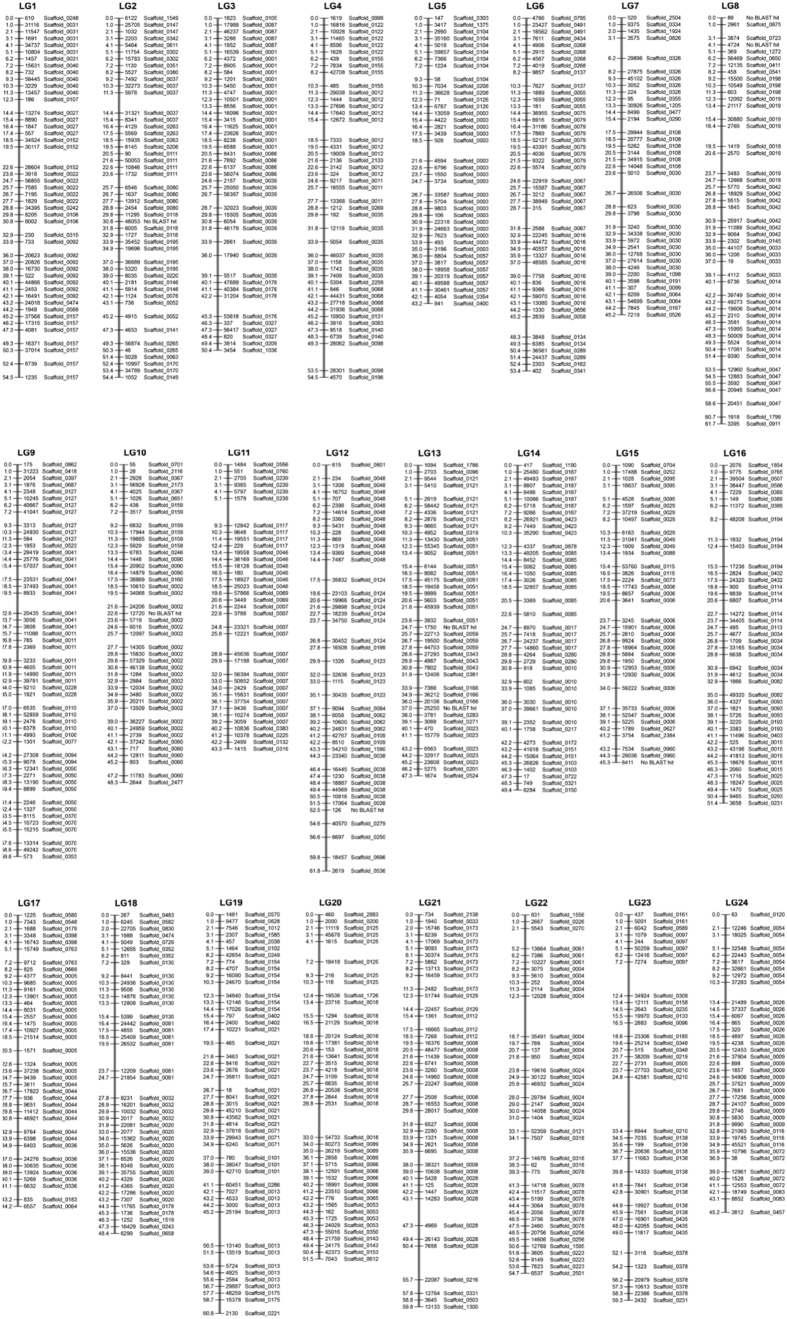
Direct comparison of *K. marmoratus* (SK) scaffolds to the genetic map constructed by 9,904 polymorphic restriction site-associated DNA (RAD)-tag (DNA markers) (Kanamori *et al*.^9^).

To construct a high-resolution genetic map, genome assemblies of the mangrove killifish were mapped to the recently established 24 linkage groups that are defined by 9,904 polymorphic restriction site-associated DNA (RAD)-tag (DNA markers)^9^. The entire set of markers of the genetic map was directly aligned to the mangrove killifish scaffolds. As result, 98% (9,726 loci) of the total markers could be assigned to scaffolds thus anchoring the genome sequence to 24 linkage groups, corresponding to the haploid chromosome number of this species (Fig. 1; Suppl. Table 4). The mean map distance ranged from 1.11 to 1.37 cM (average 1.22 cM), and the average value of cumulative number of recombination events per chromosome was 52.0 cM/LG. These numbers are like other teleosts having same number of haploid chromosomes (Suppl. Table 5)^9^.

Gene prediction in mangrove killifish genome, we used a logical pipeline (Suppl. Fig. 7) in a standard annotation approach based on a whole-genome alignment with teleost genomes and transcriptome information from RNA sequencing (RNA-seq) of different developmental stages, larvae or mixed tissues of hermaphrodites (Suppl. Table 6), resulting in a final gene set of 20,954 genes and 643 tRNAs (Table 2; Suppl. Table 7; Suppl. Fig. 8). The gene number was found to be markedly different from the RHL strain of mangrove killifish (27,328 genes and 536 tRNAs)^21^ most likely due to a higher assembly quality metrics of the SK genome. After gene annotation, total length and GC content of the SK genome reached 37 Mb and 54%, respectively (Table 2). We constructed two orthologous gene clusters, one within teleosts and one covering vertebrates from fish to human. The mangrove killifish genome contains 6,576 orthologous gene families in comparison with four teleosts, while 3,439 genes are specific to the mangrove killifish (Suppl. Fig. 9). 6,635 orthologous gene families were found after comparison of orthology relationship of mangrove killifish genome to four vertebrates with 5,415 mangrove killifish-specific gene families (Suppl. Fig. 10).

Transposable elements (TEs) are repetitive DNA sequences with the capacity to move within the genome. They are generally grouped into two classes; the class I retrotransposons which are subdivided into short interspersed elements (SINEs), long interspersed elements (LINEs), long terminal repeats (LTRs), and non-LTR retrotransposons, and the class II DNA transposons. RepeatMasker analysis of both SK and RHL strains’ assemblies showed that 27% of the genome matched to interspersed repeats (Table 3,4; Suppl. Table 8), thus approximately one-fourth of the mangrove killifish genome is composed of TEs. Teleost genomes (e.g. spotted gar, European eel, zebrafish, cod, Japanese medaka, platyfish, tilapia, stickleback, tetraodon, and fugu) show the highest diversity of TE superfamilies in vertebrates, as most TE superfamilies (e.g. Gypsy, BEL/Pao, ERV, DIRS, Penelope, Rex6/Dong, R2, LINE1, RTE, LINE2, Rex1/Babar, Jockey, Helitron, Maverick, Zisupton, Tcl-Mariner, hAT, Harbinger, PiggyBac and EnSpm) are present in all teleost genomes^24^. As noticed in other teleost genomes, TEs show a high diversity with many families present in the mangrove killifish genome (Suppl. Table 8). This diversity is also observed in the RHL strain and the composition of each TE family is quite similar in both strains (Table 4). DNA transposons (10–14%) are relatively common in two killifish genomes (Mangrove killifish and African turquoise killifish) and Japanese medaka (Atherinomorpha: Beloniformes: Adrianichthyidae), while other teleosts have considerable differences ranging from 2% for tetraodon (*Tetraodon nigroviridis*) and fugu (*Takifugu rubripes*) to 38% for zebrafish (Table 4; Suppl. Table 8)^25^. The amount of DNA transposons in the mangrove killifish genomes (10% for SK; 12% for RHL) is quite comparable to the proportion of retrotransposons (12% for SK; 10% for RHL). The most abundant DNA transposon family are the TcMar (3%) and hAT (2%) families (Suppl. Table 8). The majority of class I retrotransposons in the mangrove killifish genome are LINE elements, covering 6.4% of the genome sequence. Interestingly, the pronounced abundance of rolling-circle (RC) eukaryotic transposons (0.7% for SK; 0.6% for RHL), known as Helitrons, compared to the abundance of some of its closest phylogenetic relatives [Japanese medaka (0.03%) and African turquoise killifish (0.06%)] (Table 4) makes of an interesting case. The expression of Helitron TEs was also examined by using the RNA-seq data, and more than 70% of exons that contain those sequences were observed to be expressed (Table 5).

## Discussion

**Figure 2:**
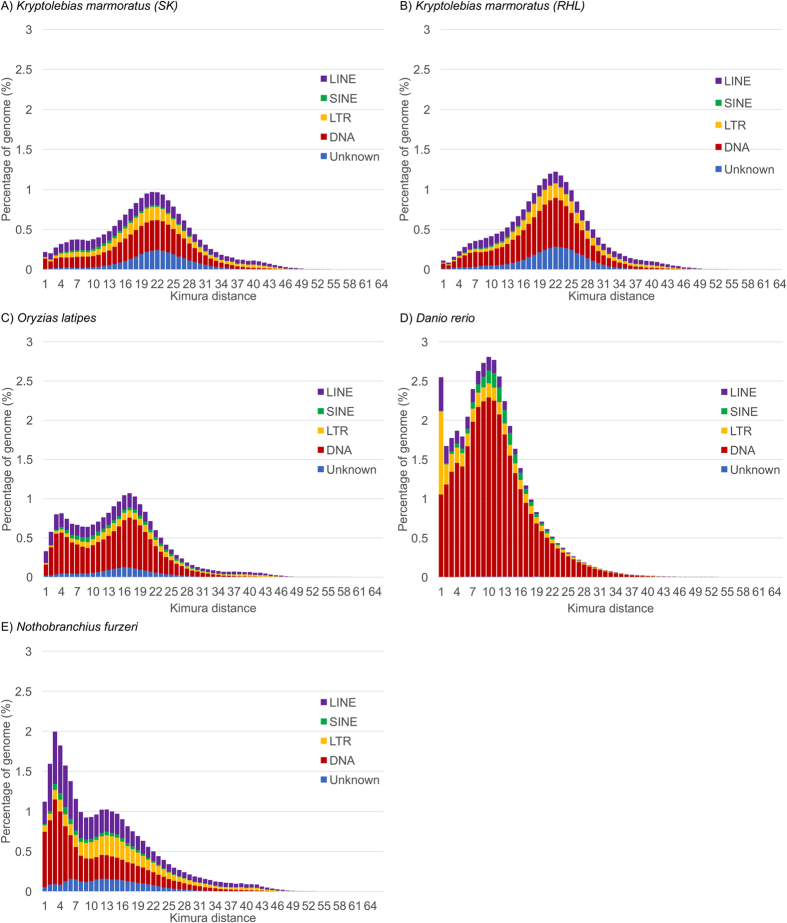
Kimura distance-based copy divergence analysis of transposable elements of (**A**) *K. marmoratus* (SK), (**B**) *K. marmoratus* (RHL), (**C**) *Oryzias latipes*, (**D**) *Danio rerio*, and (**E**) *Nothobranchius furzeri*. Y-axis represents genome coverage for each type of TEs (i.e. DNA transposons, SINE, LINE, LTR retrotransposons, and unknown TEs), and X-axis represent K-value.

**Figure 3:**
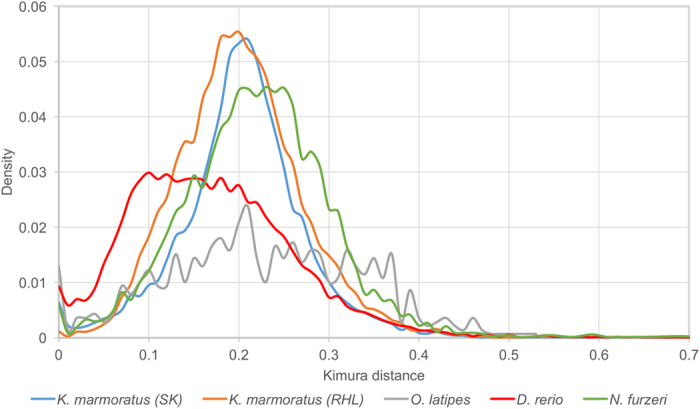
Kimura distance-based copy divergence analysis of RC/Helitrons of *K. marmoratus* (SK), *K. marmoratus* (RHL), *Oryzias latipes, Danio rerio*, and *Nothobranchius furzeri*.

Comparative analyses of Kimura distances showed that, while the two killifish genomes, Japanese medaka and African turquoise killifish all have rather recent TE copies^24^, the TEs of the mangrove killifish genomes are relatively older (Fig. 2). Similarly, TE sequence divergence relative to TE consensus sequences shows a peak at about 20% for mangrove killifish, while for the other studied fish species (namely the Japanese medaka, African turquoise killifish and the zebrafish) the divergence rate peak is lower (Fig. 3), providing a clear indication that the mangrove killifish has more diverged copies of TEs. Recently, a positive correlation between TE content and genome size was observed in teleosts^24^, and this positive correlation applies also to the mangrove killifish (Suppl. Fig. 11). In flowering plants, the transition from outcrossing to selfing is considered as a common evolutionary event^26^. Fewer members of TE classes were observed in the selfer *Arabidopsis thaliana* than in the predominantly outcrossing relative *Arabidopsis lyrata*^27^. A similar phenomenon was observed in species of the weed genus *Capsella*, although TE load comparison between selfing and outcrossing *Capsella* showed either no differences or TE enrichment in the outcrossing *Capsella*^28^. Genome analysis revealed that this was due to the accumulation of TE members in the outcrossing progenitor *Capsella grandiflora* rather than to the loss of TE members in the selfer *Capsella rubella*^28^. A recent study on several asexual lineages of arthropods and their sexual relatives noted no accumulation of TEs in the non-recombining genomes^29^. Following these observations, we may assume that an outcrossing mating system is considered to play a crucial role in driving the evolutionary dynamics of TEs. However, such a correlation of the number and composition of TE families for selfing versus outcrossing is less clear in fish. The mangrove killifish has a comparably diverse composition and high abundance of TEs as many other fish.

Approximately one-fourth of the mangrove killifish genome is comprised of TEs. Diversity and activity of mangrove killifish TEs indirectly represent its occasional outcrossing with gonochoristic males. In general, selfing induces potentially a critical impact on genome construction with a decline of internal or external TE invasion due to self-fertilization, which triggers reduced exchanges between selfers^30^,^31^. Because most teleosts employ external fertilization, their genomes can be susceptible to horizontal TE transfer^32^, resulting in higher diversity and activity of TEs. Since the mangrove killifish maintains internal self-fertilization with occasional outcrossing, several TEs can be potentially introduced by mating. In the rare case that a horizontal TE transfer occurs, once it takes place, it may be that it cannot be purged out as effectively as in the non-selfers. A previous theoretical approach explained the discrepancy of a significant amount of active TEs observed in several selfing animals that selfers might experience occasional outcrossing to hinder the eradication of TEs^33^.

The evidence of different TE diversity of the mangrove killifish compared to those of other teleosts could potentially help us understand its unique mode of reproduction. Previously, the potential effect of several TEs (i.e. Rex element and others) that directly mapped to sex chromosomes suggested their putative involvement in the process of molecular differentiation of sex chromosomes in Nile tilapia and Antarctic fish^34^,^35^,^36^. More recently, the analysis was expanded to not only the sex determination regions of the Y and W sex chromosomes but also the corresponding regions of the X and Z chromosomes in several fishes^37^. Thus, analysis of the accumulation of TEs on autosomes and/or TE-rich locus may help to explain the sexual development of the mangrove killifish after completion of genome sequencing.

By their mobility, TEs have the potential to modify genomes, but it is unclear whether their diversity or activity can explain/promote adaptation to pressure and equilibrium^38^. Although selfing will generally reduce the effective population size^39^,^40^, the observation of a high genetic diversity combined with the genetic subdivision of the population structure suggests that local populations of mangrove killifish have been relatively stable and that they did not experience major recent reductions of effective population size^41^. Of diverse possible factors, the relatively high content of RC/helitrons would contribute to the high genetic diversity of *K. marmoratus* populations. Kimura distance revealed that the genome of the mangrove killifish contains many more old RC/helitron copy than those of other killifish (Fig. 3; Suppl. Table 9). This subfamily of TEs is known to be involved in mediating duplication, shuffling, and recruitment of host genes^42^. Transposition events that affect the genome structure could have led to lineage-specific genetic diversity. Although critical evaluation of the relationship between TE diversity and ecological pressure requires further understanding of the molecular mechanisms of TEs, the here presented information on the genome and TEs in mangrove killifish is a unique reference as a self-fertilizing teleost genome and may provide an essential resource to understand teleost genome evolution, as TE diversity and abundance clearly contribute to genome evolution and adaption^43^.

## Materials and Methods

### Ethics in experiments

All animal handling and experimental procedures were approved by the Animal Welfare Ethical Committee and the Animal Experimental Ethics Committee of the Sungkyunkwan University (Suwon, South Korea). Experiments were carried out in accordance with the approved guidelines of the Animal Experimental Ethics Committee of the Sungkyunkwan University.

### Genetic background of the sequenced *K. marmoratus* specimen

*Kryptolebias marmoratus* (order Cyprinodontoformes; family Rivulidae; formerly known as *Rivulus marmoratus*; mangrove rivulus) were kindly provided by Dr William P. Davis (US EPA, Gulf Breeze, FL) and maintained exclusively by selfing. For each library preparation, total genomic DNA was extracted from a liver tissue of single hermaphroditic *K. marmoratus* and used for genomic DNA sequencing.

### Genomic DNA isolation

Liver (approximately 10 mg per individual adult hermaphrodite) was homogenized in a sterile container with gDNA isolation buffer (Tris-Cl, 10 mM, pH 8.0; NaCl, 100 mM; ethylenediaminetetraacetic acid (EDTA), 25 mM, pH 8.0; proteinase K, 100 μg/ml; sodium dodecyl sulfate (SDS), 0.5%; RNase,1 μg/ml). The sample was incubated in a water bath at 55 °C overnight. The gDNA was isolated with phenol/chloroform (Sigma, St. Louis, MO, USA) and chloroform (Sigma), and precipitated with 10 M ammonium acetate (0.2 volumes, Sigma) and isopropanol (0.5 volumes, Sigma). After washing with 70% ethanol, the gDNA was dissolved in TE (Tris-Cl, 10 mM, pH 8.0; EDTA, 1 mM) buffer and stored at 4 °C. Finally, gDNA was qualified and quantified using a spectrophotometer (Qiaxpert^®^, Qiagen, Hilden, Germany) and electrophoresis with 0.8% agarose gels.

### Pair-end sequencing

We sequenced DNA using the Illumina HiSeq 2000 platform (GenomeAnalyzer, Illumina, San Diego, CA, USA) with recommended protocols from the manufacturer. We randomly sheared 5 μg of *K marmoratus* gDNA using the nebulizer (GenomeAnalyzer, Illumina, San Diego, CA, USA) following the manufacturer’s instructions. The fragmented DNA was end-repaired using T_4_ DNA polymerase and Klenow polymerase with T_4_ polynucleotide kinase for phosphorylation of 5′ ends of the DNA. To ligate Illumina paired-end adaptor oligonucleotides with the sticky ends of DNA, a 3′ overhang was created using a 3′-5′ exonuclease-deficient Klenow fragment. Products were electrophoresed on an agarose gel, and fragments of each size were stabbed with a scalpel blade. We employed different fragment sizes to increase the genomic coverage per paired-end sequenced. DNA was enriched with Solexa primers and 18 cycle PCR reaction was performed according to the manufacturer’s instructions. Subsequently, the GenomeAnalyzer paired-end flow-cell was prepared and clusters of PCR colonies were then sequenced on the GenomeAnalyzer platform according to the manufacturer’s instructions. FASTQ sequence files were reproduced from raw images.

### Genome size estimation

In this study, genome size was calculated based on the frequency distribution analysis of k-mers with the raw sequencing read data set, as employed in previous studies^44^,^45^. The distribution profiles of the k-mer were analyzed using an out-of-core k-mer counter, meryl (http://sourceforge.net/apps/mediawiki/kmer). The sequencing depth was calculated by the formula M = N∗(L-K + 1)/L, where M is the peak depth, N is the sequencing depth, L is the average read length, and K is the k-mer length, respectively. Using a default k-mer length of 17 bases, the genome size of *K. marmoratus* was calculated as 755,646,977 bp (≈756 Mb, Suppl. Fig. 2), resulting in a similar size as the genome of the Japanese medaka (≈700 Mb).

### Assembly

In both short and long paired-end reads, duplicate, microbial, adapters, and low quality reads with at least 1 N were removed using SOAP-denovo2 program package^46^. A total of 80 Gb of genomic data that contained more than 90% of bases with base quality equal to Q20 or greater than Q20 moved for the *de novo* assembly. Before assembly of the raw reads, we excluded highly repetitive, non-informative reads, and reads, which consisted entirely of short tandem repeats. ALLPATHS‐LG (Ver. r42411) was applied with default parameters. As a result, a draft genome of 680 Mb with scaffold N50 values of 2.2 Mb (contig and scaffold statistics are in Suppl. Table 10) was obtained and quality metrics was comparable to results of other Illumina genome assemblies. The final assembly was anchored to a high-resolution genetic map constructed by 9,904 polymorphic RAD-tag (DNA markers)^23^. All marker sequences were aligned to mangrove killifish scaffolds, and scaffolds aligning to markers in the same linkage group were considered anchored.

### Assembly quality assessment

Assembly quality of the *K. marmoratus* genome was checked with Core Eukaryotic Genes Mapping Approach (CEGMA) (http://korflab.ucdavis.edu/datasets/cegma). In total 248 core, eukaryotic genes (CEGs) from *Arabidopsis thaliana, Caenorhabditis elegans, Drosophila melanogaster, Homo sapiens, Saccharomyces cerevisiae*, and *Schizosaccharomyces pombe* (listed alphabetically) were mapped on the *K. marmoratus* genome assembly. The results showed that the *K. marmroatus* genome assembly covered more than 86% of the completed CEGs and more than 97% of the partial CEGs (Suppl. Table 3).

### Gene-level synteny comparison

Gene-level synteny of the mangrove killifish genome was compared with the genomes of Japanese medaka, stickleback, and zebrafish that have published chromosome assembly information in teleosts. Briefly, entire protein sequences of the mangrove killifish were analyzed with the BLAST Reciprocal Best Hit in NCBI. Then 530 scaffolds (44.4%) containing reciprocal best-matched genes were directly mapped to the other fish genomes. All scaffolds that contained less than five genes were excluded, and the number of breakpoints in each scaffold with their proportion was calculated. Of all scaffolds, results of the longest scaffolds (scaffold#: 1, 2, 4, and 7) are presented in this manuscript.

### Repeat analysis

We used the RepeatMasker fish library together with a *de novo* generated repeat library to perform repetitive sequence analysis. To identify transposable elements (TEs) at the DNA and protein levels, homologous repeat family annotation was conducted by employing the programs RepeatMasker (ver. 4.0.5) and RepeatProteinMask (http://www.RepeatMasker.org) with default parameters against the TE database Repbase (version 20160829)^47^. The *de novo* repeat family was analyzed with RepeatModeler (ver. 1.0.8; http://www.RepeatMasker.org) using default parameters. To obtain consensus sequences from the alignments, the entire identified TEs sequences were aligned with Muscle software^48^. All TE sequences were classified with RepeatClassifier in the RepeatModeler package against Repbase^49^. Tandem repeats were also analyzed using TRFfinder (ver. 4.04) (parameters settings: match = 2, mismatch = 7, delta = 7, PM = 80, PI = 10, Minscore = 50, and MaxPeriod = 10)^50^. The above procedure was applied to all fish genomes in this study.

### RNA-seq

Three different developmental stages (stage 15, 30, and larvae) and mixed tissues (e.g. brain, gill, gonad, liver, kidney, ovary, testis, muscle) from adult hermaphrodites were homogenized in TRIZOL^®^ reagent (3 volumes, Invitrogen, Paisley, Scotland). Total RNA was isolated according to the manufacturers’ protocols. DNA digestion was performed using DNase I (Sigma). Total RNA was quantified by UV absorption at 260 nm and quality checked by analyzing the ratios A230/260 and A260/280 using a spectrophotometer (QIAxpert^®^, Qiagen, Hilden, Germany). A paired-end library was synthesized and sequenced using the Genomic Sample Preparation Kit (Illumina, San Diego, CA, USA) and Illumina HiSeq^TM^ 2000 (Illumina) according to the manufacturer’s instructions at the National Instrumentation Center for Environmental Management (NICEM, Seoul National University, Seoul, South Korea). Briefly, short fragments were isolated with the MinElute PCR Purification Kit (Qiagen, Chatsworth, CA, USA). Adaptor-ligated fragments were separated by size on an agarose gel, and the desired range of cDNA fragments (200 ± 25 bp) was excised from the gel. Suitable fragments were purified as templates for PCR amplification and subsequently, PCR amplified to create the final cDNA library template. The image data output was transformed by base calling into sequence data. Image deconvolution and quality value calculations were conducted using Illumina HCS 1.1 software based on the Illumina GA pipeline (ver. 1.6) following the protocol of the manufacturer (Illumina).

### Transcriptome assembly

Low-quality sequences (reads containing more than 50% bases with Q-value ≤ 20), adaptor-only reads, empty nucleotides (‘N’ in the end of reads), and adaptor sequences were totally removed from raw reads in the clean process. All the clean reads were subsequently assembled to generate contigs, unigenes, and non-redundant unigenes using the *de novo* assembler Trinity (ver. 2.0.6)^51^. Candidate coding regions from the assembled transcripts and/or contigs were analyzed with TransDecoder (http://transdecoder.sourceforge.net). The regions were used for BLAST analysis against the NCBI non-redundant (nr) protein database. The presence of conserved domains in the assembled transcripts was identified and annotated using InterProScan5^52^. Gene Ontology (GO) and KEGG (Kyoto Encyclopedia of Genes and Genomes) pathway analysis of the contigs were performed using Blast2GO^53^. Three main categories of GO such as cellular component, biological process, and molecular function were analyzed after comparing for similarities using default parameters at the NICEM, Seoul National University (Seoul, South Korea).

### Transposon expression analysis

The preprocessed RNA-seq reads were aligned against the scaffold assembly by using the STAR program (ver. 2.5.1b) with gene annotation data and default parameters^54^. The numbers of mapped reads in exons were counted by using the HTSeq program (ver. 0.6.1)^55^. The expression level of exons overlapping with transposons was calculated by the FPKM (Fragments Per Kilobase of transcript per Million fragments mapped) measure^56^. Three FPKM scores (1, 0.1, and 0.001) were used as a threshold to count the number of expressed exons.

## Additional Information

**Accession codes:** This Whole Genome Shotgun project has been deposited at DDBJ/ENA/GenBank under the accession LWHD00000000. The version described in this paper is version LWHD01000000. The genome data can also be accessible through G-browser (http://bioinfo.konkuk.ac.kr/cgi-bin/gb2/gbrowse/kryMar). The Transcriptome Shotgun Assembly project has been deposited at DDBJ/EMBL/GenBank under the accession # GENF00000000. The version described in this paper is the first version, GENF01000000.

**How to cite this article**: Rhee, J.-S. *et al*. Diversity, distribution, and significance of transposable elements in the genome of the only selfing hermaphroditic vertebrate *Kryptolebias marmoratus.* *Sci. Rep.* **7**, 40121; doi: 10.1038/srep40121 (2017).

**Publisher's note:** Springer Nature remains neutral with regard to jurisdictional claims in published maps and institutional affiliations.

## Supplementary Material

Supplementary Tables

## Figures and Tables

**Table 1:** Assembly statistics.

Assembly methods		ALLPATHS-LG (Ver. r42411)
Scaffolds	Total length (bp)	680,349,455
	Total number	3,072
	N50 (bp)	2,229,659
	Minimum length (bp)	3,954
	Maximum length (bp)	11,911,191
	nN (%)	5.66
	GC (%)	37.76

**Table 2:** Details of the gene annotation.

#genes	Total Length (bp)	Average Length (bp)	Density (bp)	Largest (bp)	GC content (%)
20,954	37,255,075	1,778	32,469	89,619	54.05

**Table 3:** Contents and classification of repeats identified in the *K. marmoratus* SK genome.

Repeat elements	*K. marmoratus* (SK)		
	Copy number	Bases	Percent (%)
DNA	364,269	69,083,668	10.15
SINE	31,960	4,972,714	0.73
LINE	173,854	43,256,267	6.36
LTR	144,730	31,019,789	4.56
Satellite	4,252	884,548	0.13
RC/Helitron	20,430	4,437,200	0.65
Simple_repeat	234,100	8,952,672	1.32
Low_complexity	33,489	1,484,546	0.22
rRNA	1,667	296,691	0.04
Unknown	184,901	32,842,992	4.83
Total	1,193,652	185,353,175	27.24

**Table 4:** Comparison of transposable elements (TEs) in nine teleost genomes.

TE classes	DNA	LINE	LTR	SINE	RC/Helitron	Unclassified	Total
*K. marmoratus* (SK)	10.15	6.36	4.56	0.73	0.65	4.83	27.28
*K. marmoratus* (RHL)	12.06	5.64	4.11	0.34	0.57	4.41	27.13
*O. latipes*	14.09	5.22	2.75	1.2	0.03	2.07	25.37
*D. rerio*	38.27	3.61	5.86	2.41	1.48	0.25	51.89
*C. carpio*	18.57	5.57	4.66	0.61	0.79	2.41	32.61
*N. furzeri*	11.35	11.08	5.97	1.2	0.06	3.37	33.02
*T. rubripes*	2.65	3.51	2.06	0.22	0.04	0.37	8.86
*T. nigroviridis*	1.93	2.27	1	0.18	0.03	0.95	6.36
*G. aculeatus*	3.58	2.76	3.1	0.36	0.1	0.77	10.68

The numbers for each TE class represent percentage.

**Table 5:** Expression of the RC/Helitron transposable elements*.

FPKM threshold	Embryo (St. 15)	Embryo (St. 30)	Larvae	Mixed tissues (adult)
1	471 (0.75)	455 (0.73)	446 (0.71)	443 (0.71)
0.1	545 (0.87)	535 (0.85)	532 (0.85)	522 (0.83)
0.01	550 (0.88)	539 (0.86)	534 (0.85)	527 (0.84)

^*^Among exons overlapping with the RC/Helitron transposable elements (total 626 exons), the number of exons (fraction in parentheses) that were expressed in different RNA-seq samples was counted by using the FPKM measure with three thresholds.
