# Supplementary material for: Diversity, distribution, and significance of transposable elements in the genome of the only selfing hermaphroditic vertebrate Kryptolebias marmoratus
Source: Sci Rep. 2017 Jan 10;7:40121. doi: 10.1038/srep40121 (PMC5223126; doi:10.1038/srep40121)
Supplement: Supplementary Tables [file srep40121-s1.pdf]

<Scientific Reports>-revised

Diversity and distribution of transposable elements in the genome of the only selfing hermaphroditic vertebrate *Kryptolebias marmoratus*

Jae-Sung Rhee[1,#], Beom-Soon Choi[2,#], Jaebum Kim[3,#], Bo-Mi Kim[4], Young-Mi Lee[5], Il-Chan Kim[6], Akira Kanamori[7], Ik-Young Choi[8,*], Manfred Schartl[9,*], Jae-Seong Lee[4,*]

[1] Department of Marine Science, College of Natural Sciences, Incheon National University, Incheon 22012, South Korea

[2] Phyzen Genomics Institute, Seoul 08787, South Korea

[3] Department of Stem Cell and Regenerative Biology, College of Animal Bioscience & Technology, Konkuk University, Seoul 05029, South Korea

[4] Department of Biological Science, College of Science, Sungkyunkwan University, Suwon 16419, South Korea

[5] Department of Life Science, College of Natural Sciences, Sangmyung University, Seoul 03016, South Korea

[6] Division of Polar Life Sciences, Korea Polar Research Institute, Incheon 21990, South Korea

[7] Division of Biological Science, Graduate School of Science, Nagoya University, Furocho Chikusa, Nagoya 464-8602, Japan

[8] Department of Agriculture and Life Industry, College of Agriculture and Life Sciences, Kangwon National University, Chuncheon 24341, South Korea

[9] Physiological Chemistry, University of Würzburg, Biozentrum, Am Hubland, and Comprehensive Cancer Center, University Clinic Würzburg, Josef Schneider Straße 6, 98074 Würzburg, Germany, and Institute for Advanced Studies and Department of Biology, Texas A&M University, College Station, Texas 77843, USA

[#] These authors contributed equally to this work.

*Corresponding authors and equally contributing senior authors: jslee2@skku.edu (J.-S. Lee), choii@kangwon.ac.kr (I.-Y. Choi), phch1@biozentrum.uni-wuerzburg.de (Manfred Schartl)

Supplementary Table 1 Read statistics of the five libraries used in whole genome sequencing.

Type of library	Platform	Reads No	Reads length (bp)	%	Read coverage (X)	Physical coverage (X)
Short Paired-End						
Paired-End (200 bp)	Illumina	337,951,370	33,863,601,073	85.9	50	50
Paired-End (400 bp)	Illumina	361,545,328	33,675,377,620	76.6	49	106
Long Mate-Paired						
Mate-Paired (3 kb)	Illumina	92,291,244	4,573,585,805	22.9	7	203
Mate-Paired (5 kb)	Illumina	48,047,230	1,777,747,510	30.8	3	177
Mate-Paired (20 kb)	Illumina	128,010,352	6,400,517,600	28.5	9	1,882
Total			80,290,829,608		118	2,418

Supplementary Table 2 Results of the reference-assisted chromosome assembly. The Japanese medaka genome was used in assembly as reference.

Min. synteny size[1]	5 kb	10 kb	50 kb	100 kb
No. chromosome[2]	275	144	80	59
Total length[3]	633,887,021	616,992,130	571,293,273	549,026,507
Alignment coverage[4]	93	91	84	81
Max. chromosome size[5]	19,236,552	23,401,386	27,813,725	28,259,665
Min. chromosome size[6]	3,656	5,984	94,939	94,939
N50[7]	8,850,094	13,116,237	17,093,001	17,038,606
No. used scaffolds[8]	1,163	905	470	368

[1] Min. synteny size: Minimum size of synteny block
[2] No. Chromosomes: Total number of chromosome after assembly
[3] Total Length: total length of entire chromosome
[4] Alignment Coverage: Percentage ratio of total length of targeted genomic region to entire scaffolds
[5]Maximum chromosome size
[6]Minimum chromosome size
[7]Chromosome N50
[8]No. used scaffolds in the assembly

Supplementary Table 3 Core eukaryotic genes (CEGs) evaluated for the completeness of the *K. marmoratus* genome assembly based on 248 CEGs.

	#Prots	%Completeness	#Total	Average	%Ortholog
Complete	219	88.31	290	1.32	25.57
Group 1	58	87.88	71	1.22	18.97
Group 2	47	83.93	58	1.23	21.28
Group 3	53	86.89	73	1.38	26.42
Group 4	61	93.85	88	1.44	34.43
Partial	247	99.6	412	1.67	44.53
Group 1	65	98.48	101	1.55	38.46
Group 2	56	100	84	1.5	41.07
Group 3	61	100	113	1.85	49.18
Group 4	65	100	114	1.75	49.23

Prots: number of 248 ultra-conserved CEGs present in genome
%Completeness: percentage of 248 ultra-conserved CEGs present
Total: total number of CEGs present including putative orthologs
Average: average number of orthologs per CEG
%Ortholog: percentage of detected CEGS that have more than 1 ortholog

Supplementary Table 4 Numbers of *K. marmoratus* scaffolds assigned to the genetic map constructed from 9,904 polymorphic restriction site-associated DNA (RAD)-tag (DNA markers) (Kanamori et al., 2016).

Linkage groups (GSs)	Number of markers assigned to scaffolds	Number of anchored scaffolds	Total length of anchored scaffolds (bp)
LG1	534	50	43,583,539
LG2	492	97	45,332,593
LG3	472	42	30,945,331
LG4	458	75	47,009,385
LG5	445	49	27,453,993
LG6	428	56	33,022,072
LG7	415	77	34,573,799
LG8	409	60	36,227,512
LG9	417	54	39,504,894
LG10	412	44	37,654,586
LG11	410	59	27,681,848
LG12	401	57	27,195,155
LG13	400	49	33,291,728
LG14	401	49	26,645,211
LG15	396	63	37,389,495
LG16	390	69	46,488,007
LG17	395	45	33,077,976
LG18	382	55	35,384,646
LG19	391	51	28,753,773
LG20	379	34	35,667,507
LG21	365	61	37,518,447
LG22	369	48	34,452,709
LG23	305	57	18,130,879
LG24	260	13	23,299,616
Total	9,726	1,213[a]	635,003,388[b]

[a]If a scaffold is anchored in more than two LGs, then it is counted as one when the total count is calculated.

[b]If a scaffold is anchored in more than two LGs, then its length is summed only once when the total length is calculated.

Supplementary Table 5 Average value of recombination events per chromosome in teleosts. The information was updated from a previous study (Kanamori et al., 2016).

Fish	Number of chromosomes	Average value of cumulative number of recombinations/chromosome (cM/chromosome)
Oryzias latipes[a]	24	56.5
Amphilophus spp.[b]	24	59.5
Hippoglossus hippoglossus[c]	24	63.1
Oreochromis niloticus L.[d]	24	49.0
Xiphophorus maculatus[e]	24	55.3
Kryptolebias marmoratus[f]	24	52.0

[a] Naruse *et al.* (2000)
[b] Recknagel *et al.* (2013)
[c] Palaiokostas *et al.* (2013a)
[d] Palaiokostas *et al.* (2013b)
[e] Amores *et al.* (2014)
[f] This study

Supplementary Table 6 Read statistics of RNA-seq reads from *K. marmoratus* samples used for the transcriptome assembly.

	Reads No.	Reads Length	Clear No.	Clear Length
Embryo (St. 15)	181,978,852	17,725,598,107	136,593,829	13,104,412,892
Embryo (St. 30)	62,154,962	6,050,406,139	49,149,637	4,718,983,873
Larvae	58,165,274	5,656,594,441	46,033,299	4,411,594,363
Mixed tissues (adult)	52,860,064	5,141,375,789	42,617,022	4,088,000,090
Entire genes			20,954	70,624,330

Supplementary Table 7 tRNA statistics.

tRNAs encoding standard 20 AA	466
Selenocysteine tRNAs (TCA)	5
Possible suppressor tRNAs (CTA,TTA)	1
tRNAs with undetermined/unknown isotypes	3
Predicted pseudogenes	168
Total tRNAs	643

Supplementary Table 8 Classifications and frequencies of transposable elements and other repeats

Classes	Copies	Bases	Percent (%)
**DNA transposons**			
DNA/Academ	4	282	0.00
DNA/CMC	9,520	1,187,916	0.17
DNA/Crypton	1,688	170,482	0.03
DNA/Dada	524	59,460	0.01
DNA/Ginger	524	46,774	0.01
DNA/hAT	61,486	11,102,647	1.63
DNA/IS3EU	552	93,874	0.01
DNA/Kolobok	1,265	265,711	0.04
DNA/Maverick	1,472	136,336	0.02
DNA/Merlin	695	53,850	0.01
DNA/MULE	788	67,829	0.01
DNA/P	351	93,925	0.01
DNA/PIF	28,516	5,209,602	0.77
DNA/PiggyBac	1,165	124,833	0.02
DNA/Sola	264	25,170	0.00
DNA/TcMar	85,390	20,791,224	3.06
DNA/Zisupton	1,997	151,197	0.02
DNA/other	168,068	30,166,851	4.43
**Retrotransposons**			
SINE/5S	14	1,026	0.00
SINE/L2	12	691	0.00
SINE/MIR	6,637	1,298,746	0.19
SINE/tRNA	16,205	2,571,468	0.38
SINE/other	9,092	1,133,898	0.17
LINE/Dong	6,884	1,869,167	0.27
LINE/I	4,071	1,229,816	0.18
LINE/L1	7,306	3,192,940	0.47
LINE/L2	38,315	9,677,670	1.42
LINE/Penelope	12,652	2,179,830	0.32
LINE/Proto2	204	134,490	0.02
LINE/R2	793	136,017	0.02
LINE/Retroposon	17	1,285	0.00
LINE/Rex	10,638	2,886,152	0.42
LINE/RTE	34,556	9,729,273	1.43
LINE/other	58,418	12,332,586	1.81
LTR/Bhikhari	26	2,859	0.00
LTR/DIRS	1,354	424,922	0.06
LTR/ERV1	1,452	262,055	0.04
LTR/ERVK	35	2,420	0.00
LTR/Gypsy	9,663	5,328,268	0.78
LTR/Ngaro	10,627	1,772,925	0.26
LTR/Pao	1,276	973,046	0.14
LTR/Retroposon	54	11,872	0.00
LTR/other	119,922	21,990,597	3.23
Satellite	4,252	884,548	0.13
RC/Helitron	20,430	4,437,200	0.65
Simple_repeat	234,100	8,952,672	1.32
Low_complexity	33,489	1,484,546	0.22
rRNA	1,667	296,691	0.04
Unknown	184,901	32,842,992	4.83
**Total**	**1,193,652**	**185,353,175**	**27.24**

Supplementary Table 9 Comparison of RC/Helitron copies in five teleost genomes.

RC/Helitron	Total count	Total length (bp)	Percent of genome (%)
K. marmoratus (PAN)	20,430	4,463,882	0.65
K. marmoratus (DAN)	18,207	3,712,091	0.57
O. latipes	1,387	235,577	0.03
D. rerio	82,361	20,114,621	1.48
N. furzeri	6,677	727,066	0.06

Supplementary Table 10 Assembly statistics.

	Contig	Scaffold
Total length (bp)	621,459,966	680,349,455
No.	34,712	3,072
N50 (bp)	33,074	2,229,659
Min length (bp)	2,000	3,954
Max length (bp)	385,258	11,911,191

Supplementary Figure 1. Estimation of genome size of *Kryptolebias marmoratus (SK)* based on distribution of 17 k-mer frequency in raw sequencing reads.

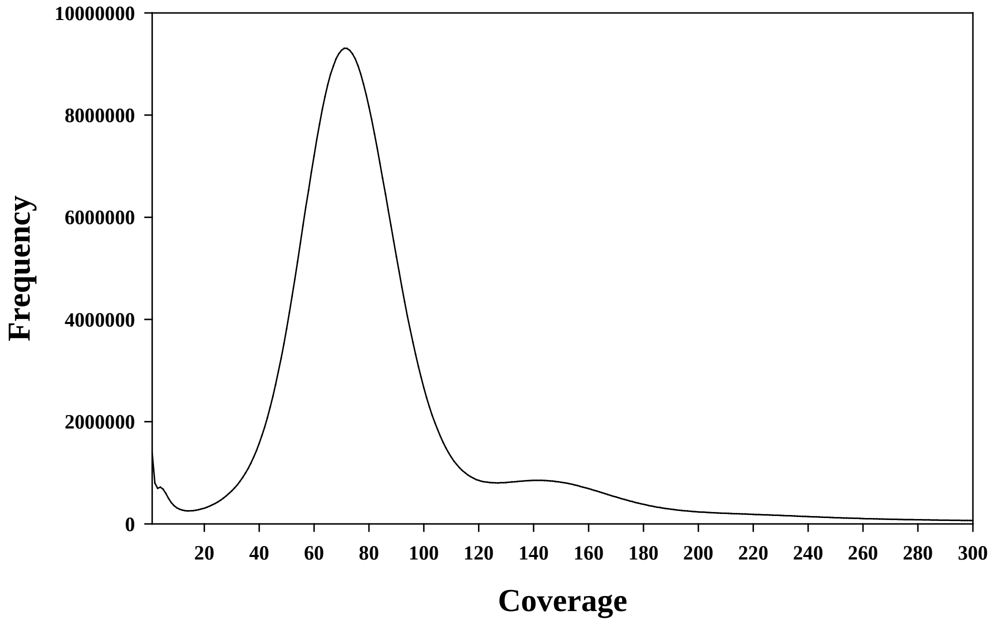

Supplementary Figure 2. Mapping of *Danio rerio titin A* and *B* gene clusters against *K. marmoratus (SK)* assemblies. Both zebrafish titin clusters were mapped to a single scaffold (#0294) of *K. marmoratus (SK)*.

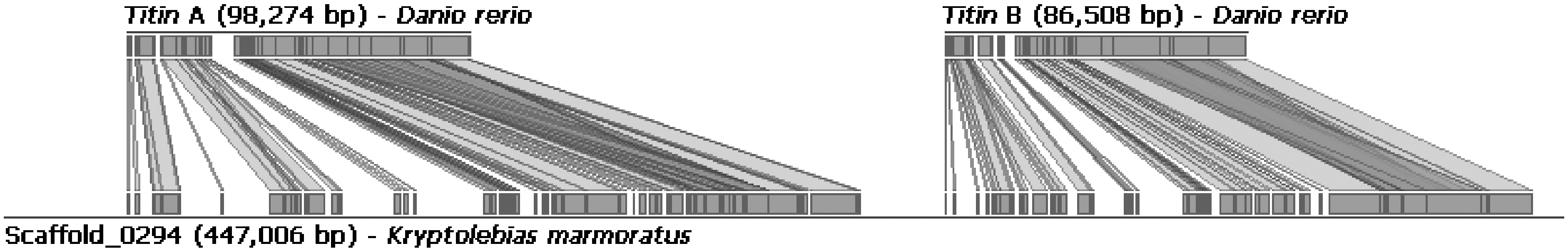

Supplementary Figure 3. Mapping of *Oryzias latipes* major histocompatibility complex (*MHC*) class I clusters against *K. marmoratus (SK)* assemblies. The Japanese medaka *MHC* class I region was mapped to a single scaffold (#0104) of *K. marmoratus (SK)*.

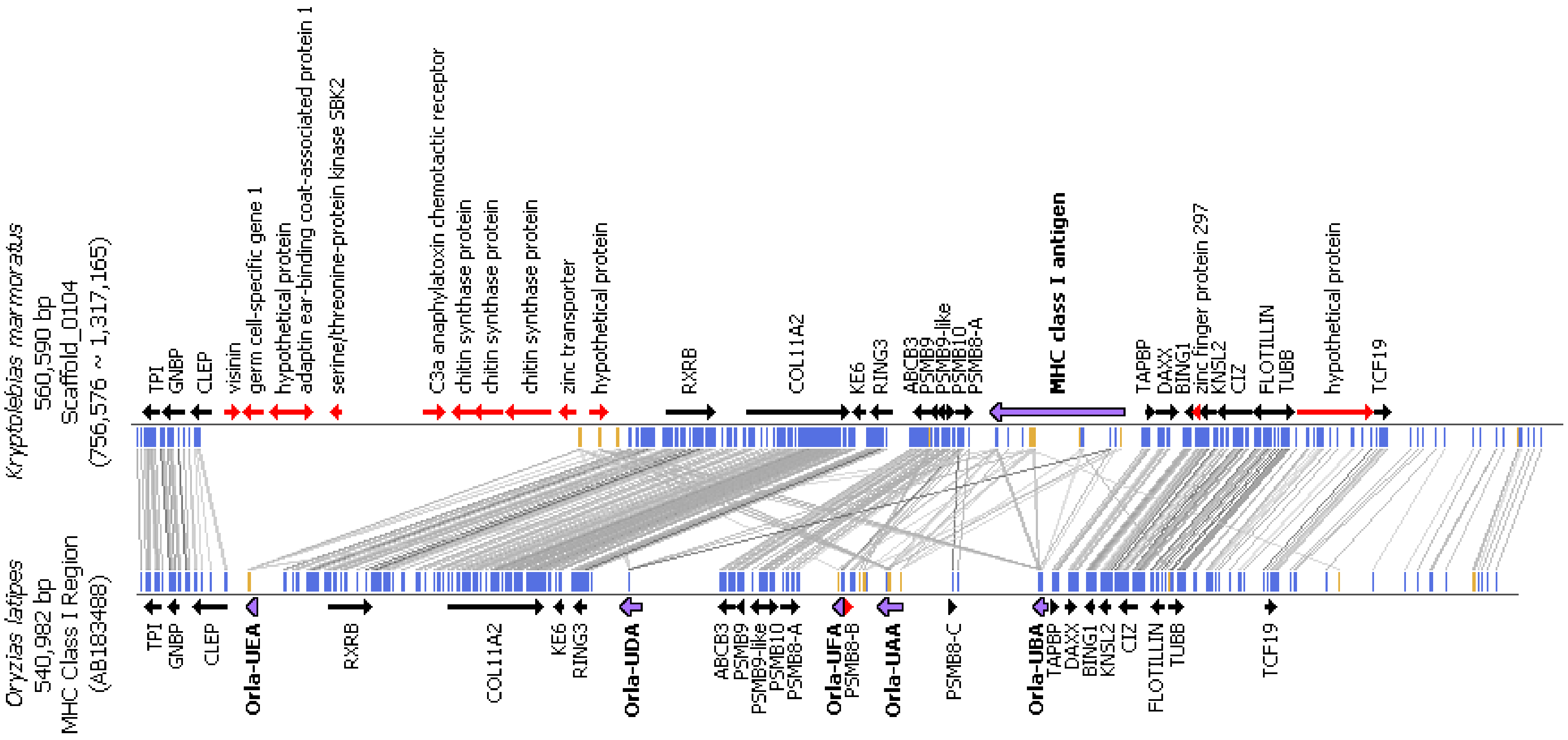

Supplementary Figure 4. A) Seven intact *Hox* gene clusters identified in *K. marmoratus (SK)* assemblies. B) Phylogeny of *Hox* gene clusters of 9 teleosts. Figure reproduced with permission from Kim et al. (2016).

A)

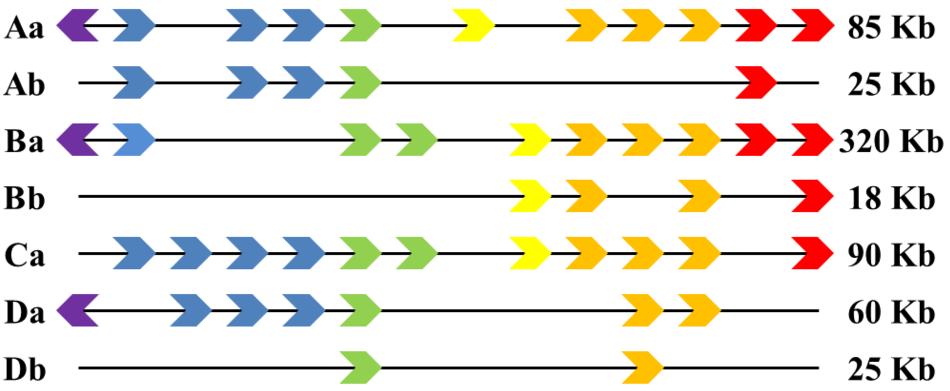

B)

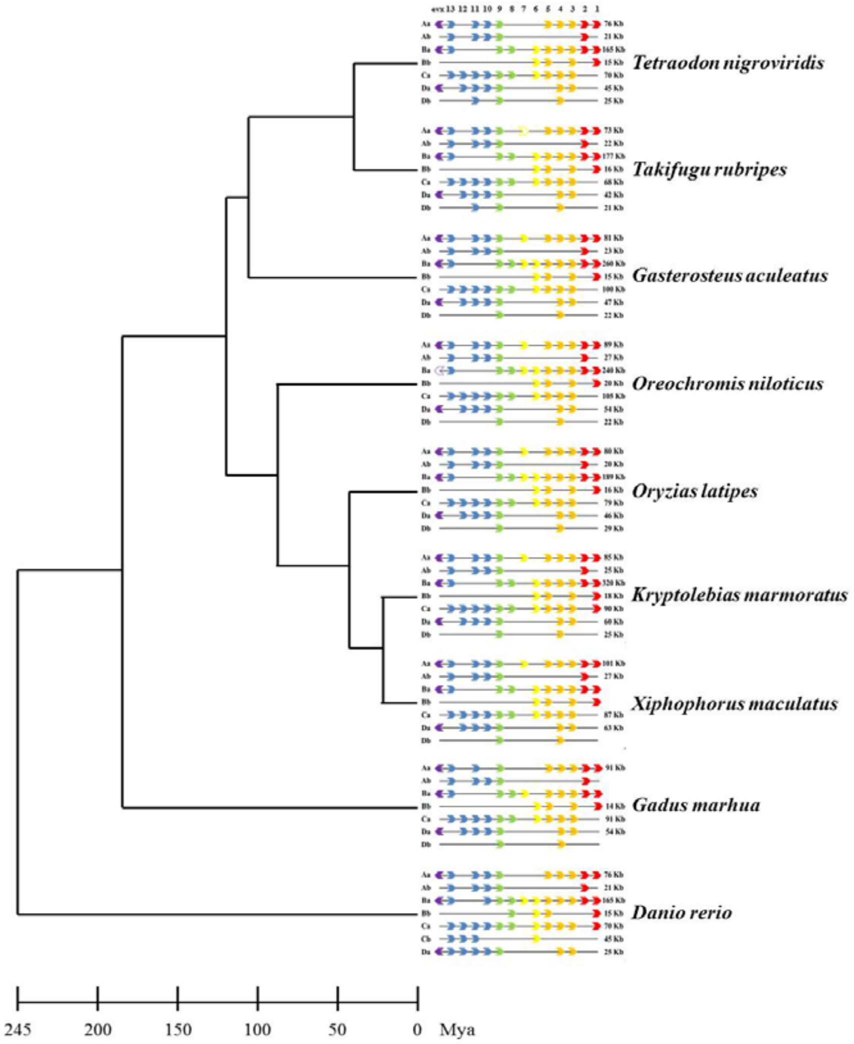

Supplementary Figure 5. Comparison of gene level synteny using direct mapping of filtered *K. marmoratus (SK)* scaffolds to other teleost genomes (medaka, stickleback, and zebrafish)

A) Overall numbers of breakpoints in filtered scaffolds and their proportions.

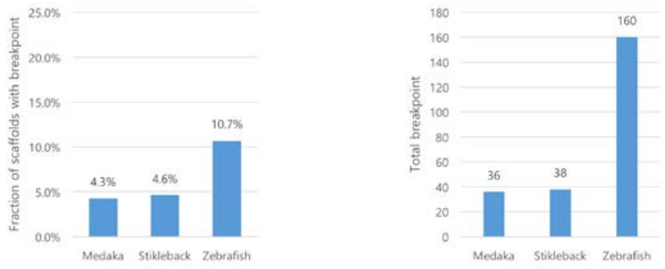

B) Example of scaffold_0001

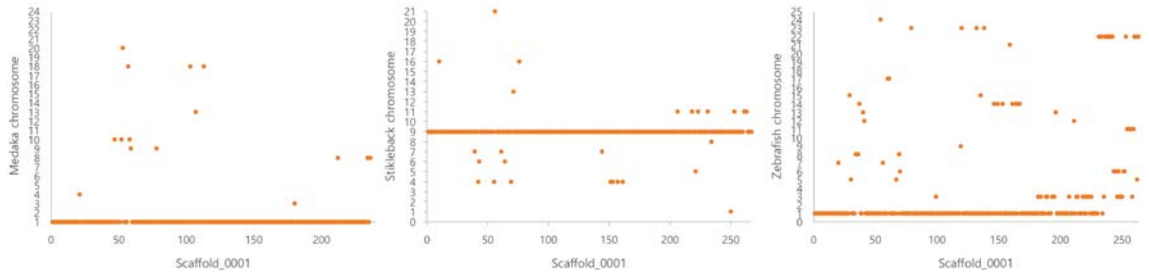

C) Example of scaffold_0002

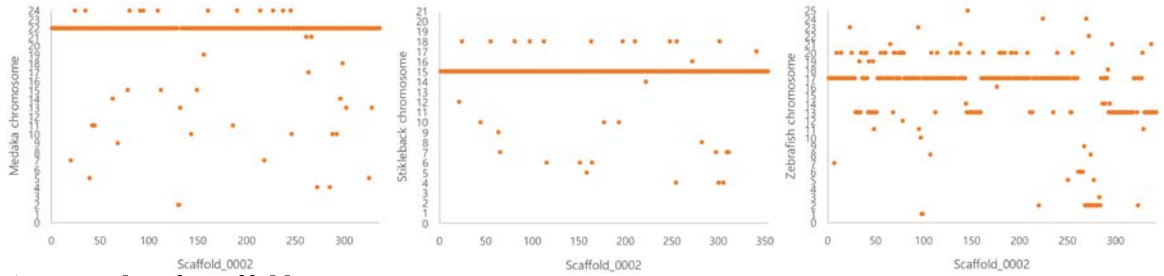

D) Example of scaffold_0003

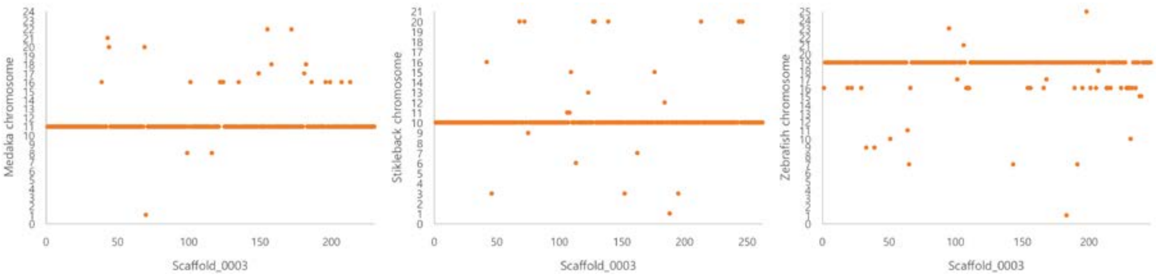

E) Example of scaffold_0004

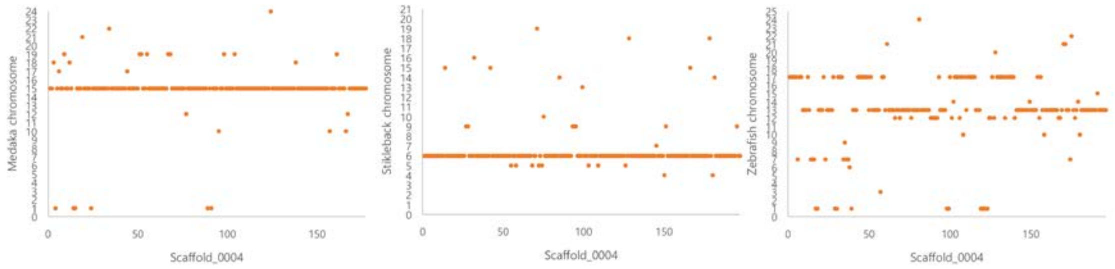

Supplementary Figure 6. GC content and sequencing depth. The GC content and average sequencing depth was analyzed by 500 bp non-overlapping sliding window along the genome assemblies. The box plot was performed using the R package.

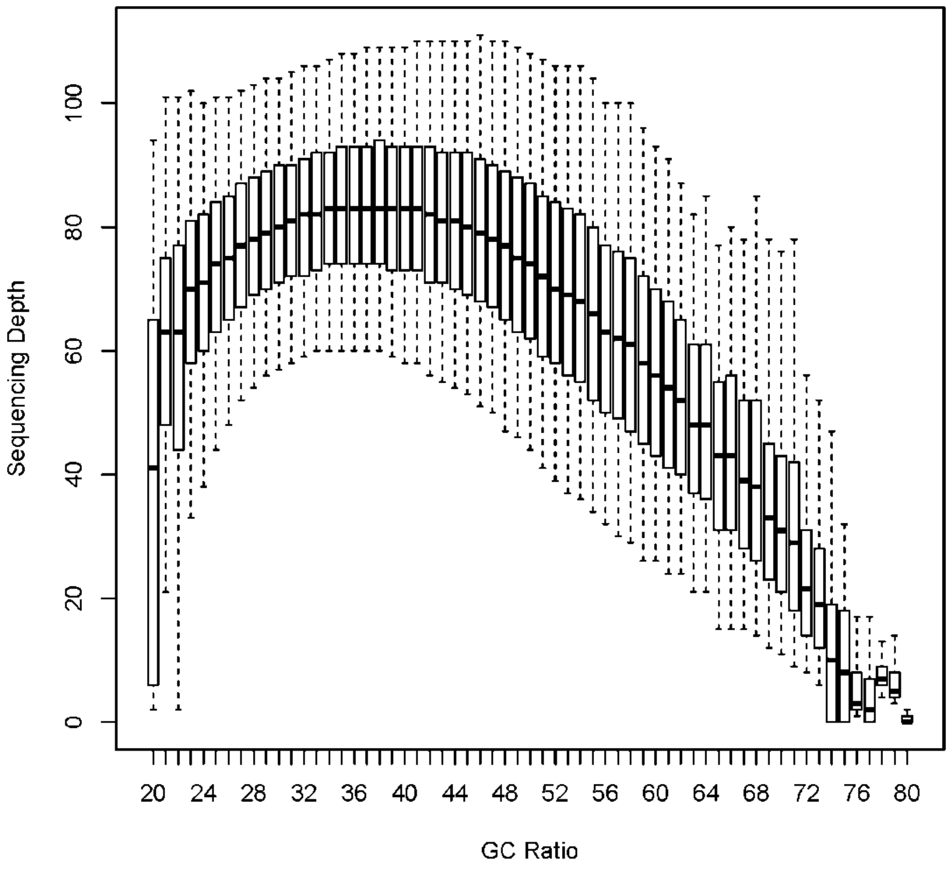

Supplementary Figure 7. Pipeline employed for gene prediction.

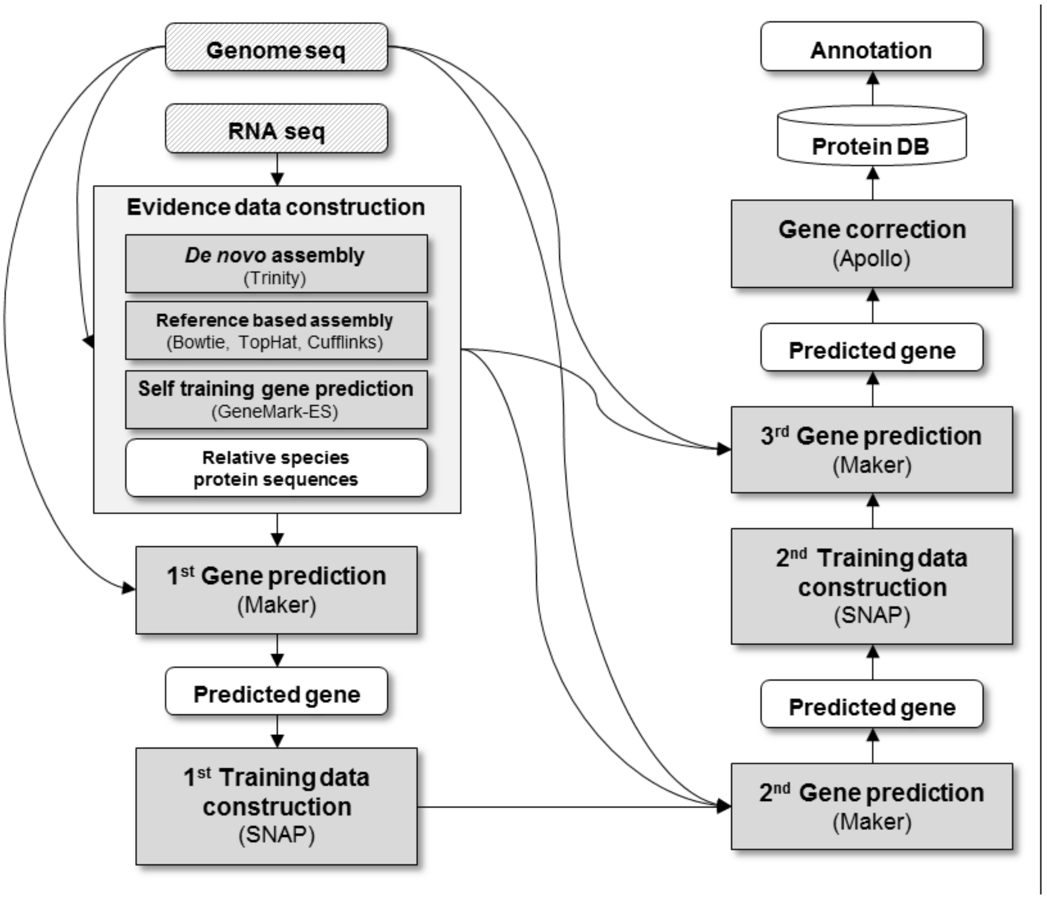

Supplementary Figure 8. Gene length distribution.

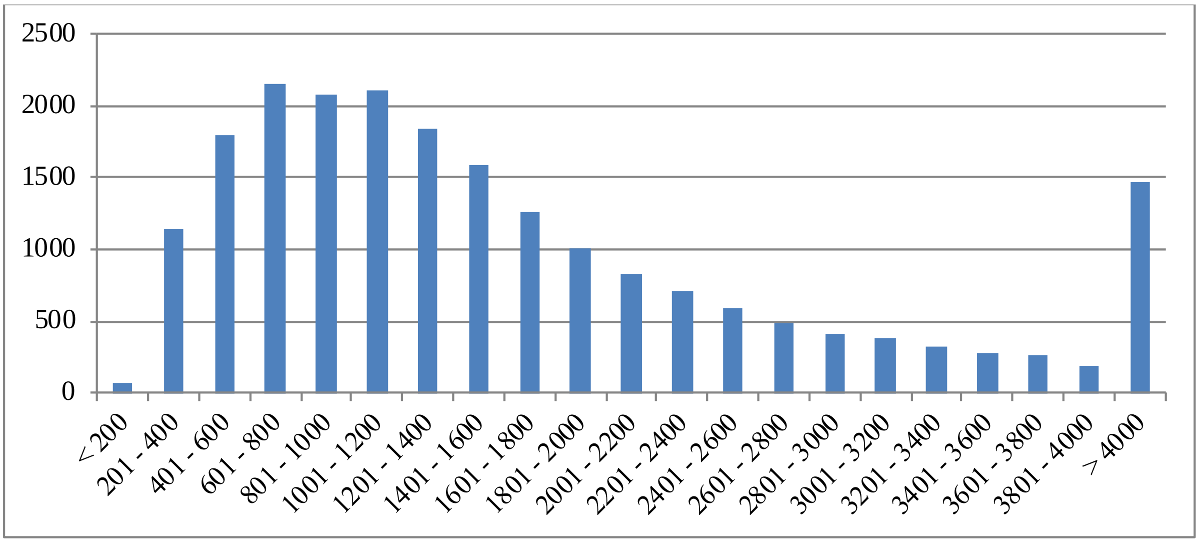

Supplementary Figure 9. Orthologous gene clusters within teleosts.

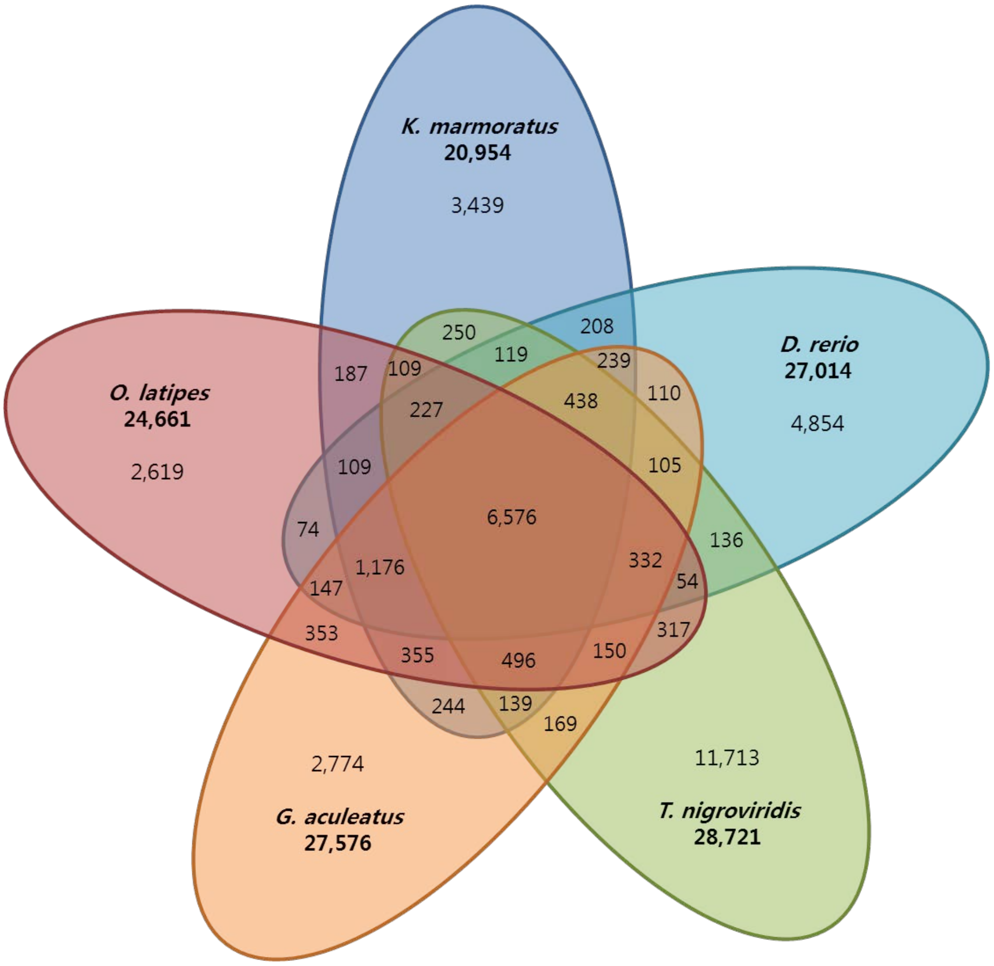

Supplementary Figure 10. Orthologous gene clusters within vertebrates.

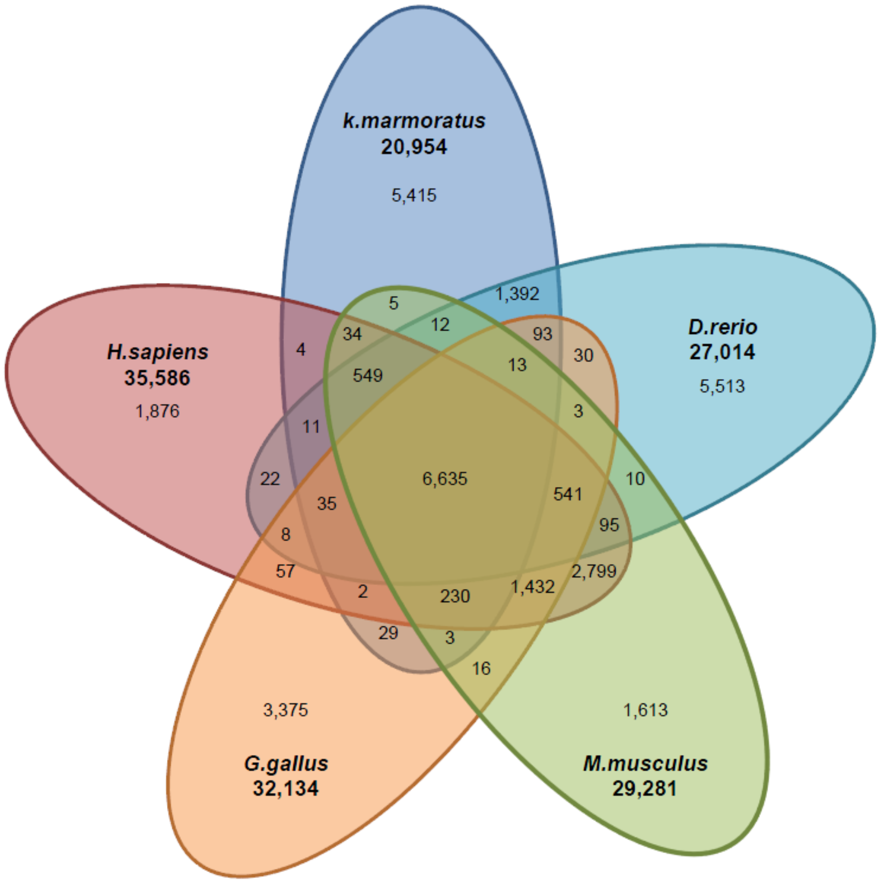

Supplementary Figure 11. Correlation between genome size and transposable element (TE) content in teleosts. Informations on genome size and TE content of Cod, Fugu, Medaka Platyfish, Stickleback, Tetraodon, Tilapia, and Zebrafish were adopted from Chalopin et al. (2015). The information of Carp was adopted from Xu et al. (2014).

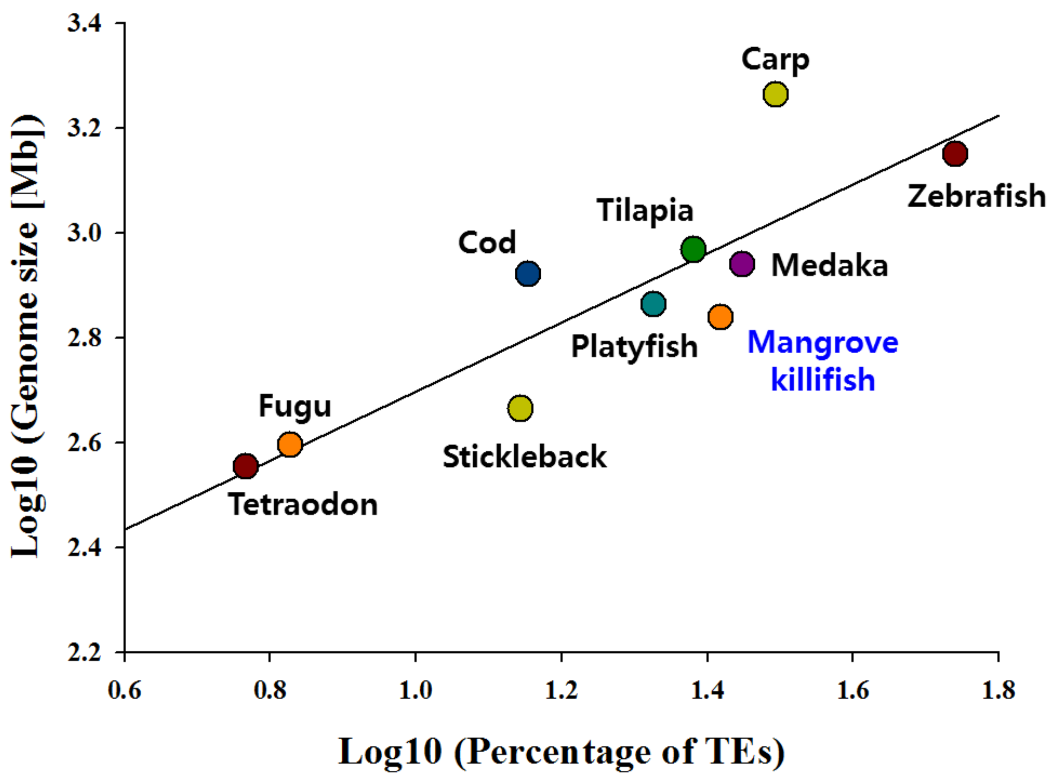

References

Amores, A., Catchen, J., Nanda, I., Warren, W., Walter, R., Schartl, M., Postlethwait, J.H., 2014. A RAD-tag genetic map for the platyfish (*Xiphophorus maculatus*) reveals mechanisms of karyotype evolution among teleost fish. Genetics 197, 625-641.

Chalopin, D., Naville, M., Plard, F., Galiana, D., Volff, J.N., 2015. Comparative analysis of transposable elements highlights mobilome diversity and evolution in vertebrates. Genome Biol. Evol. 7, 567-580.

Kanamori, A., Sugita, Y., Yuasa, Y., Suzuki, T., Kawamura, K., Uno, Y., Kamimura, K., Matsuda, Y., Wilson, C.A., Amores, A., Postlethwait, J.H., Suga, K., Sakakura, Y., 2016. A genetic map for the only self-fertilizing vertebrate. G3 6, 1095-1106.

Kim, B.-M., Lee, B.-Y., Lee, J.-H., Rhee, J.-S., Lee, J.-S., 2016. Conservation of *Hox* gene clusters in the self-fertilizing fish *Kryptolebias marmoratus* (Cyprinodontiformes, Rivulidae). J. Fish Biol. 88, 1249-1256.

Naruse, K., Fukamachi, S., Mitani, H., Kondo, M., Matsuoka, T., *et al.*, 2000. A detailed linkage map of medaka, *Oryzias latipes*: comparative genomics and genome evolution. Genetics, 154, 1773-1784.

Palaiokostas, C., Bekaert, M., Davie, S., Cowan, M.E., Oral, M., et al., 2013a. Mapping the sex determination locus in the Atlantic halibut (*Hippoglossus hippoglossus*) using RAD sequencing. BMC Genomics 14: 566.

Palaiokostas, C., Bekaert, M., Khan, M.G., Taggart, J.B., Gharbi, K., et al., 2013b. Mapping and validation of the major sex-determining region in Nile tilapia (*Oreochromis niloticus* L.) using RAD sequencing. PLoS One, 8, e68389.

Recknagel, H., Elmer, K.R., Meyer, A., 2013. A hybrid genetic linkage map of two ecologically and morphologically divergent Midas cichlid fishes (*Amphilophus spp.*) obtained by massively parallel DNA sequencing (ddRADSeq). G3, 3, 65-74.

Xu, P., Zhang, X., Wang, X., Li, J., Liu, G., Kuang, Y., Xu, J., Zheng, X., Ren, L., Wang, G., Zhang, Y., Huo, L., Zhao, Z., Cao, D., Lu, C., Li, C., Zhou, Y., Liu, Z., Fan, Z., Shan, G., Li, X., Wu, S., Song, L., Hou, G., Jiang, Y., Jeney, Z., Yu, D., Wang, L., Shao, C., Song, L., Sun, J., Ji, P., Wang, J., Li, Q., Xu, L., Sun, F., Feng, J., Wang, C., Wang, S., Wang, B., Li, Y., Zhu, Y., Xue, W., Zhao, L., Wang, J., Gu, Y., Lu, W., Wu, K., Xiao, J., Wu, J., Zhang, Z., Yu, J., Sun, X., 2014. Genome sequence and genetic diversity of the common carp, *Cyprinus carpio*. Nat. Genet. 46, 1212-1219.
